# Using an Electronic Immunization Registry (Aplikasi Sehat IndonesiaKu) in Indonesia: Cross-Sectional Study

**DOI:** 10.2196/53849

**Published:** 2025-03-27

**Authors:** Dewi Nur Aisyah, Astri Utami, Fauziah Mauly Rahman, Nathasya Humaira Adriani, Fiqi Fitransyah, M Thoriqul Aziz Endryantoro, Prima Yosephine Hutapea, Gertrudis Tandy, Logan Manikam, Zisis Kozlakidis

**Affiliations:** 1 Department of Epidemiology and Public Health Institute of Epidemiology and Health Care University College London London United Kingdom; 2 Digital Transformation Office Ministry of Health Republic of Indonesia Jakarta Indonesia; 3 Aceso Global Health Consultants Pte Limited Singapore Singapore; 4 Department of Public Health Monash University Tangerang Indonesia; 5 School of Computer Science Faculty of Engineering University of Sydney Sydney Australia; 6 Directorate of Immunization Management Ministry of Health Republic of Indonesia Jakarta Indonesia; 7 International Agency for Research on Cancer World Health Organization Lyon France

**Keywords:** immunization, registry, digital, puskesmas, public health center, mobile app

## Abstract

**Background:**

Electronic immunization registries (EIRs) are being increasingly used in low- and middle-income countries. In 2022, Indonesia’s Ministry of Health introduced its first EIR, named Aplikasi Sehat IndonesiaKu (ASIK), as part of a comprehensive nationwide immunization program. This marked a conversion from traditional paper-based immunization reports to digital routine records encompassing a network of 10,000 primary health centers (*puskesmas*).

**Objective:**

This paper provides an overview of the use of ASIK as the first EIR in Indonesia. It describes the coverage of the nationwide immunization program (Bulan Imunisasi Anak Nasional) using ASIK data and assesses the implementation challenges associated with the adoption of the EIR in the context of Indonesia.

**Methods:**

Data were collected from primary care health workers’ submitted reports using ASIK. The data were reported in real time, analyzed, and presented using a structured dashboard. Data on ASIK use were collected from the ASIK website. A quantitative assessment was conducted through a cross-sectional survey between September 2022 and October 2022. A set of questionnaires was used to collect feedback from ASIK users.

**Results:**

A total of 93.5% (9708/10,382) of public health centers, 93.5% (6478/6928) of subdistricts, and 97.5% (501/514) of districts and cities in 34 provinces reported immunization data using ASIK. With >21 million data points recorded, the national coverage for immunization campaigns for measles-rubella; oral polio vaccine; inactivated polio vaccine; and diphtheria, pertussis, tetanus, hepatitis B, and *Haemophilus influenzae* type B vaccine were 50.1% (18,301,057/36,497,694), 36.2% (938,623/2,595,240), 30.7% (1,276,668/4,158,289), and 40.2% (1,371,104/3,407,900), respectively. The quantitative survey showed that, generally, users had a good understanding of ASIK as the EIR (650/809, 80.3%), 61.7% (489/793) of the users expressed that the user interface and user experience were overall good but could still be improved, 54% (422/781) of users expressed that the ASIK variable fit their needs yet could be improved further, and 59.1% (463/784) of users observed sporadic system interference. Challenges faced during the implementation of ASIK included a heavy workload burden for health workers, inadequate access to the internet at some places, system integration and readiness, and dual reporting using the paper-based format.

**Conclusions:**

The EIR is beneficial and helpful for monitoring vaccination coverage. Implementation and adoption of ASIK as Indonesia’s first EIR still faces challenges related to human resources and digital infrastructure as the country transitions from paper-based reports to electronic or digital immunization reports. Continuous improvement, collaboration, and monitoring efforts are crucial to encourage the use of the EIR in Indonesia.

## Introduction

### Background

Immunization is an important aspect of maintaining the health of individuals and society as a whole. It is a life-saving measure and a highly cost-effective public health intervention, an indispensable element of primary health care that shields children from avoidable mortality, illnesses, and disabilities caused by highly transmissible diseases [1]. Routine immunization plays an important role in reducing the incidence of various infectious diseases such as diphtheria, *Haemophilus influenzae* type B, measles, polio, rubella, and tetanus [2]. Located off the coast of Southeast Asia as the largest archipelagic country with 5 main islands, Indonesia has the fourth largest population in the world at >277 million [3]. This large population potentially poses the risk of rapid disease transmission if they are not protected by immunization. To ensure universal access to immunization services, vaccines must be distributed to geographically isolated regions as well as culturally or socially distinct populations, including hard-to-reach groups such as displaced individuals; migrants; and those impacted by conflict, political instability, and natural disasters [4]. Ensuring comprehensive immunization coverage for the entire population is of utmost importance yet challenging [3].

In recent years, Indonesia has faced challenges in achieving complete basic immunization, with 2020 and 2021 rates reaching 84.2%, falling short of the coverage targets of 92.9% and 93.6%, respectively [5,6]. Catch-up immunization for measles-rubella (MR) in toddlers also had declining coverage, standing at 65.3% (of a target of 76.4%) in 2020 and 58.5% (of a target of 81%) in 2021 [7]. Even with the increase in complete basic immunization coverage in 2022 (94.6%), immunization for preventable diseases remained low in low-coverage areas [5]. The COVID-19 pandemic significantly contributed to this decline as it led to disruptions in community immunization services, hindering the further attainment of herd immunity [5,6,8,9]. To address this issue, the Indonesian Ministry of Health (MoH) introduced the nationwide catch-up immunization campaign, which was called Bulan Imunisasi Anak Nasional (BIAN), in 2022 focusing on improving immunization coverage.

The MoH periodically conducts these nationwide catch-up immunization campaigns [7]. In 2022, Indonesia focused on conducting BIANs for two main activities: (1) supplementary immunization for MR and (2) catch-up immunization for children aged <5 years [7]. This focus was chosen based on several recommendations [7]. First, the National Committee for Measles-Rubella and Congenital Rubella Syndrome Elimination of Indonesia recommended to accelerate the achievement of MR and congenital rubella syndrome elimination targets in Indonesia by strengthening routine MR immunization with doses 1 and 2, aiming for a minimum coverage of 95%. Second, the National Immunization Technical Advisory Group recommended implementing inactivated polio vaccine (IPV) catch-up immunization for infants and children who had missed their scheduled IPV immunization to close immunity gaps and provide protection against the polio virus type 2. Third, the expert committee on diphtheria control recommended catch-up immunization efforts to close immunity gaps, especially among children aged <5 years (toddlers), and outbreak response immunization in areas experiencing outbreaks using appropriate vaccines for the target age group.

The BIAN was held initially for 1 month in May 2022 for the Sumatra, Kalimantan, Sulawesi, Nusa Tenggara, Maluku, and Papua islands and in August 2022 for the Java and Bali islands [7]. A total of 4 vaccines (oral polio vaccine [OPV]; IPV; MR; and diphtheria, pertussis, tetanus, hepatitis B, and *H influenzae* type B [DPT-HB-Hib]) were selected to support Indonesia’s commitment to control global diseases, such as polio eradication, elimination of MR and congenital rubella syndrome and hepatitis B, control of diphtheria, reduction of the incidence of tuberculosis, and elimination of maternal and neonatal tetanus [7]. During the implementation of this BIAN, for the first time, Indonesia used an electronic immunization registry (EIR) capturing individual-level records [5] and replacing paper-based records as the latter were shown previously to include inaccuracies due to inexact, incomplete data and late reporting [10-12].

### Objectives

EIRs as part of an immunization information system are a tool designed to provide information on immunization programs’ target populations. By definition, EIRs include information that facilitates active search to identify individuals’ vaccination data and vaccine history and provide support for determining which individuals need to be vaccinated and monitoring of immunization dropouts [13]. The 2 main databases needed for EIRs are demographic data and vaccination event data, which aim to identify the vaccine recipient and the vaccination event itself [13]. EIRs have been widely used in high-income countries as such digital reporting tools can improve reporting timeliness, precision, and overall performance [14]. Indonesia continues to improve its digital health sector and has launched the digital health transformation blueprint, aiming to create an integrated and sustainable health system [15]. As part of this effort, the government embarked on digitizing immunization reporting, shifting from a manual collection of aggregate data to individual digital records using the Aplikasi Sehat IndonesiaKu (ASIK). This paper aims to provide an overview of ASIK and its use and specifically evaluate ASIK implementation during the nationwide immunization campaign (BIAN) period in Indonesia.

## Methods

### ASIK Overview

ASIK is an integrated application system developed by the MoH of Indonesia for health care workers to capture health care services provided for communities outside public health centers (*puskesmas*) [16,17]. This application is designed to help health care workers in primary care settings record patient data, report health care services provided for each individual, input routine health screening data, and monitor patient conditions [16,17].

**Figure 1:**
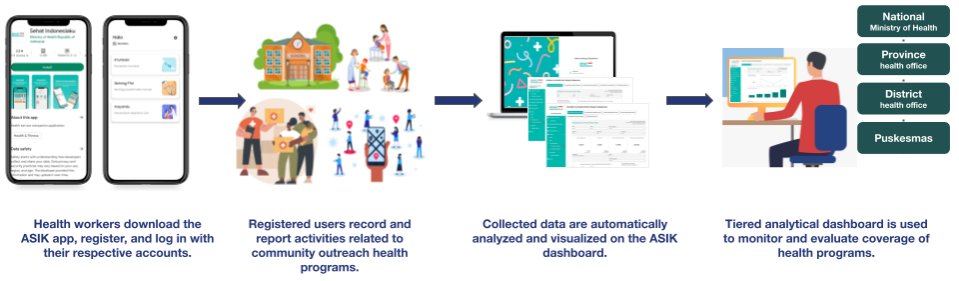
Health service program reporting scheme using Aplikasi Sehat IndonesiaKu (ASIK). Puskesmas: Indonesian for "primary health center.".

The ASIK mobile app is available in an Android version and can be accessed through the Google Play Store. Targeted users of ASIK are health care workers in primary care settings. Health care workers need to register their account, as shown in Figure 1. Once registered and verified, health care workers can report any health care services provided outside the public health centers using ASIK to record individual data. Data reported through the ASIK mobile app are collected and automatically presented in an analytical dashboard provided in a web version of ASIK [17]. This web version of ASIK can only be accessed using a predetermined username and password for public health centers, district health offices, and provincial health offices to monitor and evaluate the coverage of health care services in their respective areas. The data presented in the dashboard are automatically synchronized from the public health centers level up to the national level. A simplified flow for the use of ASIK is shown in Figure 1.

ASIK was designed in line with elements of ideal EIRs according to the Pan American Health Organization’s definition [18]. These elements encompass (1) inclusion of all persons at birth or as early as possible; (2) unique ID (national ID; biometrics or birth registration; or a unique combination of variables such as names, date or place of birth, and parental names or IDs); (3) information about each person, including information on geographical area of residence; (4) information about the vaccines administered, dates, and provider; (5) aggregation of data by geographical level as required; (6) timely individualized follow-up of vaccination schedule; (7) data entry as close to vaccination as possible (time and place); and (8) data security and protection of patient confidentiality.

The elements of ideal EIRs in ASIK were incorporated as follows: (1) allowing health workers to record individuals as soon as they are born with no restriction on age; (2) allowing health workers to record unique identification using the Indonesia resident registration number and a combination of name and date of birth as mandatory variables; (3) allowing health workers to record information about each individual using place of birth and current area of residence, which includes province name, city and district name, subdistrict name, village name, and detailed address; (4) allowing individuals to record information about vaccination, which includes vaccine name, vaccine batch number, date of vaccination, and location of vaccination; (5) a tiered dashboard that displays aggregated and structured data in spatial format from the national level to the village level; and (6) protection of patient confidentiality by ensuring a verification process during user registration and a limited access to a dashboard that displays detailed individual vaccination information (this access is designed specifically for the public health centers).

### BIAN Immunization Reporting Using ASIK

The MoH launched and tested ASIK during the BIAN in May 2022 [19]. The BIAN 2022 immunization campaign was held in various mass public facilities such as *posyandu* (community-based health care service in Indonesia where local volunteers [cadres], midwives, and health workers provide essential maternal and child health services, including immunization); public health centers; subsidiary public health centers; hospitals; clinics; immunization service posts in schools, Islamic schools (*madrasas*), and Islamic boarding schools (*pesantrens*); and other strategic community-gathering places that could be turned into immunization posts [7]. Through ASIK, health care workers were able to record immunization data individually [16]. All immunization records from ASIK were monitored by the immunization program coordinators in public health centers in near real time.

Several databases served as the sources of ASIK data, including the public health center registry (Aplikasi Registrasi Puskesmas), the MoH Data and Information Center database, the Electronic Logistic Management and Information System (SMILE), and the Ministry of Home Affairs database. The Aplikasi Registrasi Puskesmas provides information regarding the location of immunization services, including names and locations of public health centers along with registered codes for each health facility. ASIK used data from the MoH Data and Information Center to acquire information on the number of targeted immunization participants for every district and city. Integration with the SMILE database allowed ASIK to access data on the types of vaccines available, the quantity of vaccine doses, and the distribution locations. During the process of recording individual data, an interconnected application programming interface with the Ministry of Home Affairs ensured data validation for citizen identification numbers and domicile information.

**Figure 2:**
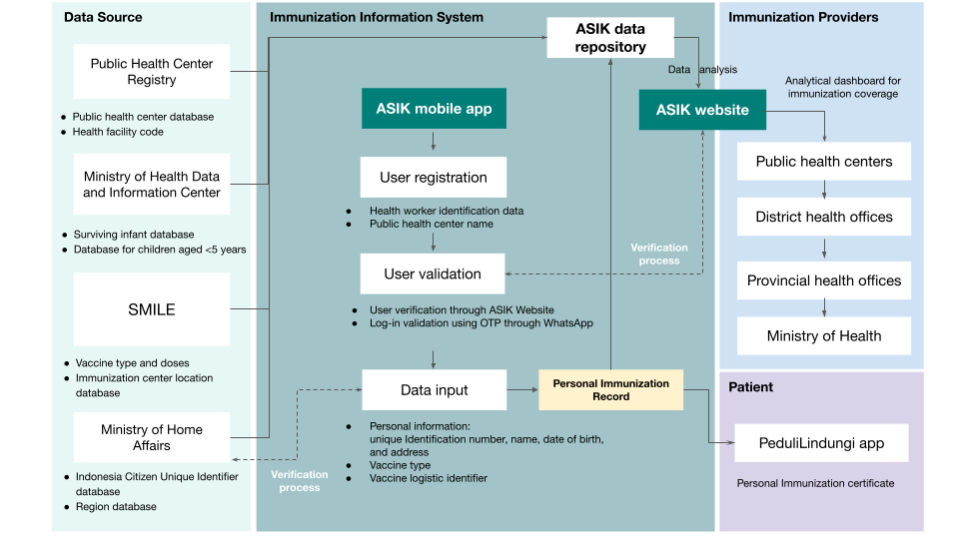
Immunization data flow in Aplikasi Sehat IndonesiaKu (ASIK). OTP: one-time password; SMILE: Electronic Logistic Management and Information System.

Each record generated through ASIK is stored in a separate database and then automatically sent to the ASIK dashboard for data analysis. Processed data are distributed to various beneficiaries, such as health facilities, district and provincial health offices, and citizens, through different platforms. In addition, individuals who have been vaccinated can receive their immunization history data through the PeduliLindungi app, which was rebranded in 2023 as SATUSEHAT Mobile [20]. Figure 2 illustrates the entire data flow within ASIK.

**Figure 3:**
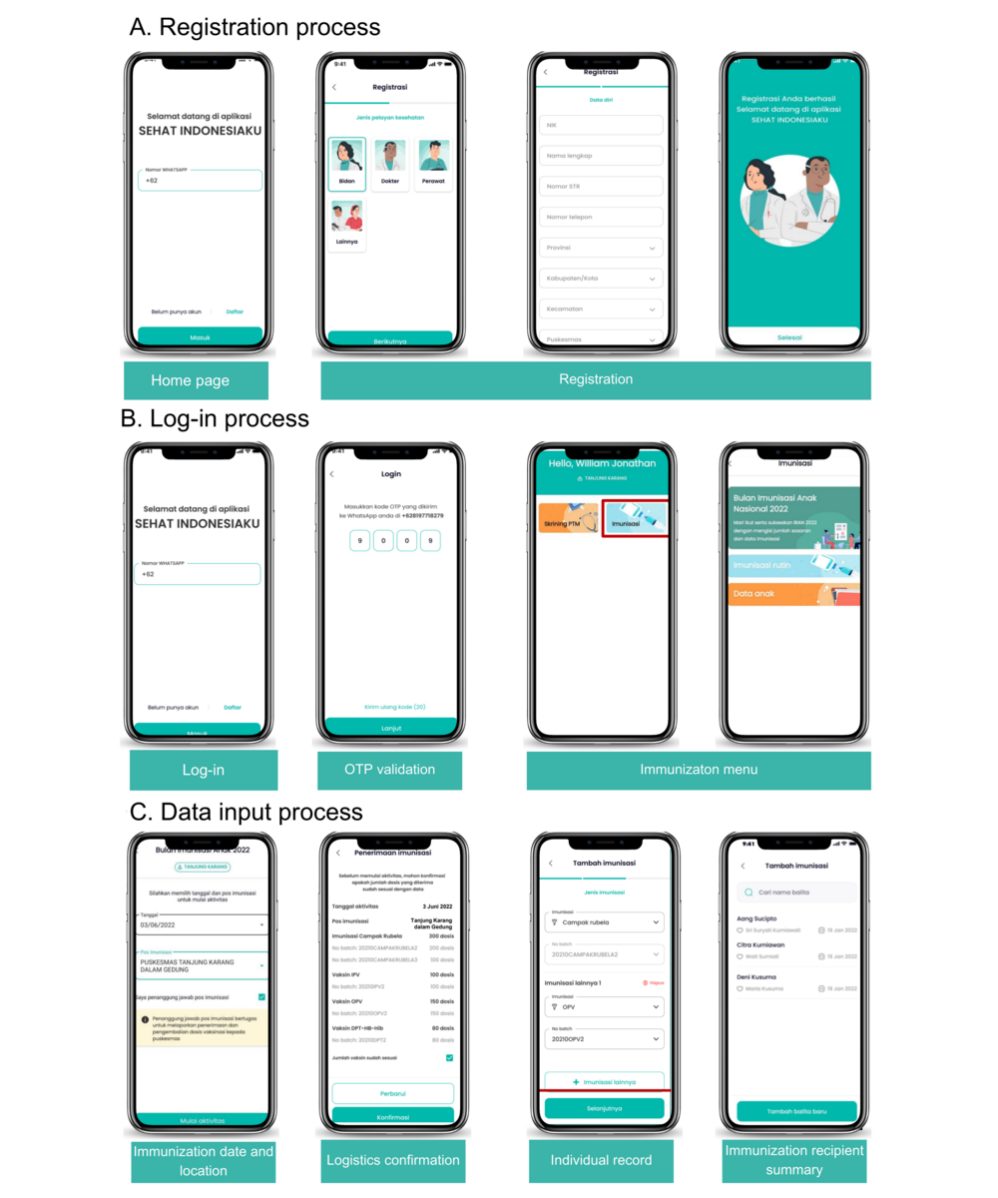
Aplikasi Sehat IndonesiaKu user interface for (A) registration process, (B) log-in process, and (C) data input process. OTP: one-time password.

The immunization data collection process in ASIK (Figure 3) comprises the steps outlined in Textbox 1.

**Immunization data collection process in Aplikasi Sehat IndonesiaKu.**

**User registration**

Vaccinators are required to undergo registration to report immunization services using ASIK. The registration process involves providing personal information, specifying the work area, and selecting a specific public health center. Upon completing the registration form, users need to input their phone numbers to receive a one-time password for app log-in. The one-time password is sent through WhatsApp to each registered user to ensure system and user security.

**Immunization data input**

Vaccinators can record immunization data at designated immunization centers. Before selecting the immunization centers, the pharmaceutical officers must report the vaccine stock distribution to the specific immunization center through the SMILE. Once the logistic stock has been reported, vaccinators can choose a specific immunization center and the date of the immunization service. They can then input immunization data, including the names and addresses of recipients and the type of vaccine administered to each individual.

**ASIK analytic dashboard**

Immunization records captured by ASIK are integrated into an analytic dashboard. Public health center immunization coordinators can monitor the results of immunization services through this dashboard. The analytic dashboard also provides a spatial representation of the data, organized at provincial, district, and city levels. Thus, immunization data from the public health centers level to the national level can be monitored in near real time, with updates every 2 hours.

**Individual immunization record**

Parents can access their children’s immunization records through the PeduliLindungi mobile app. Originally developed as a mass surveillance tool during the COVID-19 pandemic, PeduliLindungi initially presented COVID-19 vaccine certificates and later extended its functionality to include vaccine certificates from ASIK. Following the revocation of pandemic status, PeduliLindungi was further enhanced to encompass personal medical records. As a result, in 2023, the app was rebranded as a citizen health app named SATUSEHAT Mobile.

### Data Collection

Data entry is initiated by selecting the BIAN from the immunization menu within ASIK. In total, 2 distinct roles can be chosen, namely, immunization program coordinator or immunization officer. The immunization program coordinator (ie, the individual in charge of the immunization posts) validates the available vaccine doses for each immunization post to activate the individual data report feature. This involves verifying data related to logistical distribution by reporting the number of vaccine distributions and the return of vaccination doses. On the other hand, immunization officers or vaccinators focus on reporting vaccination data at the individual level.

To record individual data, either the 16 digits of the national identification number or a combination of the full name and date of birth are entered to locate the appropriate personal health record. Consequently, a list of relevant individuals appears, allowing the users to select the relevant data or create a new identification record if the data are not available. Upon completing the identification data, vaccinators proceed to enter information about the administered vaccine type. To minimize error, the system was designed to display only the list of available vaccines suitable for the individual’s age. For instance, for children aged 0 to 3 months, the system will not show the IPV in ASIK as the IPV has a minimum age requirement of 4 months. The system automatically records the results of the provided immunization services. It was recommended that vaccinators input the data immediately after services have been provided, although the option for backdating the data input is also available in ASIK, allowing users to submit delayed reports.

A quantitative survey was conducted from September 19, 2022, to October 21, 2022, using cross-sectional methods with a total of 1065 participants. All immunization program coordinators (ASIK users) from all public health centers, district health offices, and provincial health offices from 2 regions were invited to a web-based evaluation meeting. The first region represented a low data input into ASIK (the data gaps between manual and electronic reporting were approximately 30,000 to >100,000 immunization record data), and the second region represented the highest data input into ASIK (data gaps were of <10%). A total of 17.1% (182/1065) of the participants were provincial and district health officers, and 82.9% (883/1065) of the participants were health care workers from the public health centers. A questionnaire was used to collect feedback from the ASIK users (Multimedia Appendix 1). The survey used the Mentimeter platform, which enabled participants to provide direct answers and showed the cumulative, anonymized results directly. The questionnaire consisted of four sections: (1) overall user feedback on the system, (2) data reporting process, (3) supporting infrastructure, and (4) data completeness.

### Data Analysis

Three outcomes were assessed in this study: (1) ASIK implementation coverage, (2) BIAN vaccination coverage (MR, OPV, IPV, and DPT-HB-Hib immunization), and (3) ASIK use feedback from users. ASIK implementation coverage and BIAN immunization coverage were calculated from the ASIK database. BIAN vaccination coverage was presented as percentage data; it was calculated by dividing the number of vaccinations (numerator) by the targeted population number for each vaccination (denominator) at the province level.

The results of BIAN immunization services were automatically presented in a structured dashboard that was accessible for public health centers, district health offices, provincial health offices, and the MoH. These data can be queried using predetermined variables. For instance, the analysis includes metrics such as the coverage of children administered the MR vaccine, OPV, IPV, and DPT-HB-Hib vaccine by comparing the service outcome rates (ie, the number of vaccinated children compared to the target numbers). The target estimates were calculated based on the 2022 Health Development Program Target Population data [7]. Data analysis of the results of immunization services was also presented in a spatial format at the provincial, district, and city levels. Trends in service outcomes were depicted using time-series graphs. Moreover, comparative results of immunization coverage were generated (eg, by sex and age groups).

ASIK implementation coverage was measured by calculating how many public health centers used ASIK for the nationwide immunization program. The coverage was presented as percentage data, calculated by dividing the number of public health centers that reported BIAN immunization services using ASIK (numerator) by the total number of public health centers in Indonesia (denominator). The information was presented in the dashboard using the monitoring and evaluation feature. This feature enables relevant stakeholders to monitor the compliance of public health centers in reporting BIAN immunization services via ASIK. The monitoring and evaluation feature also provides information that represents the coverage of subdistricts, districts, and cities that have submitted their reports through ASIK. In addition, the dashboard generates a list of public health centers that have yet to submit their reports through ASIK. As a result, the provincial, district, and city health offices can effectively plan and implement a tiered monitoring and evaluation process.

ASIK use was assessed using a quantitative survey to obtain users’ feedback. The outcome of the quantitative survey was to measure users’ feedback on ASIK and how data collection using the digital tool was carried out, including (1) overall user feedback on the system (user understanding, user acceptance based on user interface [UI] and user experience [UX] and the variables presented, and user-friendliness of the system), (2) data reporting implementation (number of staff members for data input, flow of data reporting, and time to input data into ASIK), (3) infrastructure (mobile phone ownership and internet access), and (4) data completeness (availability of data on vaccinated individuals, comprising ID number, full name, date of birth, and gender). Outcomes 2, 3, and 4 were also explored to understand the real-life situations that potentially affected the use of an electronic reporting system in public health centers. The questions in the survey were developed as closed-ended questions, which allowed respondents to choose from a set of predefined answers quickly. The quantitative survey was analyzed using descriptive analysis. The data analysis was conducted using SPSS (version 20; IBM Corp) to describe users’ feedback regarding the mobile app use in 2 different regions.

### Ethical Considerations

The data collected for this paper do not require ethics approval in accordance with the Indonesian MoH *National Guideline and Standards for Ethical Research and Development in Health* guideline (2017, Chapter IIIB, point 2) [21] as no individual-level data are presented. The immunization analysis data are in anonymized, aggregate format and were handled following the aforementioned guidelines. For the survey, this research was conducted under the ethics approval of the International Agency for Research on Cancer Ethics Committee (22–37). Anonymous participation followed verbal informed consent, which was considered an indication of consent for obtaining and analyzing the data. There was no compensation provided for participants as it is part of the MoH immunization campaign program. No individual data were presented; only aggregated anonymized data were used as per the aforementioned guidelines.

## Results

### BIAN Immunization Reports by Province and Vaccine Type

The BIAN was held in 2 phases. The first phase began in May 2022 and ran until the end of July 2022, targeting 27 million children across 26 provinces in the Sumatra, Kalimantan, Sulawesi, Nusa Tenggara, Maluku, and Papua islands. The second phase took place in August 2022 in the Java and Bali islands (7 provinces), aiming for roughly 10 million children. The data analysis presented in this paper depicts the BIAN reports between May 2022 and December 2022. Different areas had distinct vaccination targets for each vaccine (Multimedia Appendix 2).

According to the data records in ASIK, there were 21.89 million BIAN immunization services recorded, with details on 18.3 million for the MR vaccine (18,301,057/36,497,694, 50.1%), 938,000 for the OPV (938,623/2,595,240, 36.2%), 1.2 million for the IPV (1,276,668/4,158,289, 30.7%), and 1.37 million for the DPT-HB-Hib vaccine (1,371,104/3,407,900, 40.2%).

The highest coverage of MR immunization was attained by Banten, reaching 96.8% (901,465/931,740), followed by Central Java (1,996,112/2,069,562, 96.5%) and East Java (1,797,337/2,352,409, 76.4%). In contrast, Aceh reported the lowest coverage (93,127/1,444,337, 6.4%), followed by Papua (72,368/792,523, 9.1%) and Central Sulawesi (104,316/708,642, 14.7%).

For OPV immunization, 2 provinces surpassed 100% coverage, namely, Lampung (49,833/37,827, 131.7%) and Banten (111,255/105,771, 105.2%), followed by Central Java (115,771/131,724, 87.9%). A significant contrast emerged in OPV coverage, with Papua (951/118,227, 0.8%), East Kalimantan (658/26,294, 2.5%), and Central Kalimantan (2857/58,389, 4.9%) showing the lowest coverage.

For IPV immunization, Banten reached the highest coverage (163,826/173,701, 94.3%), followed by Bali (1254/1426, 87.9%). In contrast, Papua (1814/167,678, 1.1%), East Kalimantan (747/67,544, 1.1%), and Central Kalimantan (1892/95,243, 2%) recorded the lowest IPV coverage.

For DPT-HB-Hib immunization, 2 provinces surpassed 100% coverage, namely, Banten (188,804/172,619, 109.4%) and Bali (3733/3620, 103.1%), followed by Lampung (64,043/71,504, 89.6%). Conversely, Papua (1294/194,510, 0.7%), Aceh (7282/184,475, 3.9%), and Central Sulawesi (2041/43,691, 4.7%) recorded the lowest coverage (Multimedia Appendix 3).

In summary, significant regional disparities in immunization coverage are apparent based on ASIK data. The Java and Bali islands exhibited the highest coverage across all vaccine types (MR: 7,544,342/9,435,097, 80%; OPV: 637,763/1,102,238, 57.9%; IPV: 927,925/1,747,414, 53.1%; DPT-HB-Hib: 1,087,268/1,759,356, 61.8%), whereas the Sulawesi island had the lowest coverage for 3 vaccine types (MR: 1,480,388/4,270,493, 34.7%; IPV: 25,270/406,208, 6.2%; DPT-HB-Hib: 28,745/315,809, 9.1%). Low coverage was also observed in the Papua, Maluku, and Nusa Tenggara islands for all vaccine types.

Using the specified BIAN 2022 campaign targets as benchmarks [7], we compared the immunization coverage achieved in each province with their predetermined targets. The immunization coverage report (Multimedia Appendix 4) illustrates that only 2 provinces, Banten and Lampung, successfully attained the coverage goal for MR immunization. In parallel, 4 provinces (Bali, Banten, Central Java, and Lampung) exceeded the target for OPV immunization. Similarly, the immunization target for IPV was met only by Bali, Banten, and Central Java. The DPT-HB-Hib immunization target was accomplished only by Bali, Banten, Central Java, and Lampung. These data are in line with past reports in which immunization targets were often unmet in the context of Indonesia, although the current technological implementation allowed for more granular data and for a quicker comparison at a national scale.

### Digital Versus Paper-Based Recording of BIAN Implementation

Although the implementation of the BIAN and the release of ASIK happened simultaneously, the technical implementation guideline document for BIAN stated that manual or paper-based reports are still possible using a specified format [7]. Hence, all provinces involved also recorded data using a paper-based format. This manual data collection focuses on reporting aggregate numbers of immunization services, such as the total number of children aged 12 to 59 months receiving DPT-HB-Hib immunization. The data presented in the following paragraphs reflect the paper-based recording of the same campaign used to compare the digital versus paper-based recording of BIAN implementation.

Thus, according to the official letter from the MoH regarding the achievements of the BIAN phase I and phase II in 2022 and using the data from the paper-based records alone, the immunization coverage in Indonesia for MR, OPV, IPV, and DPT-HB-Hib was 72.7% (26,529,596/36,497,639), 54.2% (1,330,928/2,454,340), 45.8% (1,842,869/4,024,564), and 61% (2,011,057/3,294,942), respectively [22] (Multimedia Appendix 3).

The highest coverage of MR immunization was attained by East Java, reaching 100.6% (2,365,820/2,352,401), followed by Jakarta (710,757/715,786, 99.3%) and Banten (918,323/931,739, 98.6%). Conversely, Aceh reported the lowest coverage for MR (280,017/1,444,335, 19.4%), followed by Papua (307,466/792,523, 38.8%) and Riau (880,867/1,913,263, 46%).

In terms of OPV immunization, 3 provinces surpassed 100% coverage, namely, Bali (786/732, 107.4%), Central Java (113,325/106,194, 106.7%), and Banten (107,570/105,085, 102.4%). A significant contrast emerged in OPV coverage, with West Nusa Tenggara (10,224/129,413, 7.9%), West Kalimantan (6556/78,005, 8.4%), and Central Kalimantan (5472/58,389, 9.4%) showing the lowest coverage.

Regarding IPV immunization, Yogyakarta attained the highest coverage at 92.2% (400/434), followed by West Java (611,346/664,213, 92%) and East Java (195,047/212,128, 91.9%). Conversely, Papua (5521/167,678, 3.3%), East Kalimantan (2744/67,544, 4.1%), and Central Kalimantan (4378/95,243, 4.6%) recorded the lowest IPV coverage.

For DPT-HB-Hib immunization, South Sumatra led with the highest coverage (36,117/36,397, 99.2%), followed by Bali (3606/3639, 99.1%) and Central Java (293,073/297,231, 98.6%). In contrast, Papua (9676/194,510, 5%), Aceh (25,323/195,289, 13%), and West Kalimantan (8510/79,635, 10.7%) reported the lowest DPT-HB-Hib coverage (Multimedia Appendix 3).

On the basis of the table in the Multimedia Appendix 3, there were gaps between manual reporting data as compared to the digital recording via ASIK.

For MR immunization, the average reporting gap was 24.4% ±3.72% (95% CI: 20.68%-28.12%); the highest gap was observed in Maluku (47.4%), followed by South Sulawesi (47.3%) and Central Sulawesi (47.3%). The greatest reporting alignment was observed in Central Java (1.5%), followed by Banten (1.9%) and South Kalimantan (11.6%).

For OPV immunization, the average gap was 18.3% ±7.26% (95% CI: 11.04%-25.56%); the highest discrepancy was observed in East Java (70.4%), followed by Gorontalo (60.6%) and the Riau islands (53.6%). The greatest alignment was observed in Lampung (−39.7%), followed by Banten (−2.8%) and Jakarta (−1.9%).

For IPV immunization, the average gap was 16.1% ±6.42% (95% CI: 9.68%-22.52%); the highest data discrepancy was observed in the Special Region of Yogyakarta (77.4%), followed by East Java (70.8%) and the Riau islands (56.5%). The closest reporting alignment was observed in Jakarta (−1.8%), followed by West Nusa Tenggara (1.6%) and Papua (2.2%).

For DPT-HB-Hib immunization, the average gap was 20.7% ±7.23% (95% CI: 13.47%-27.93%); the highest data reporting discrepancy was observed in the Riau islands (82.4%), followed by East Java (71.6%) and Maluku (58.2%). The greatest reporting alignment was observed in Banten (−12.2%), followed by Bali (−4%) and Lampung (−0.4%).

### ASIK Use and Coverage

**Figure 4:**
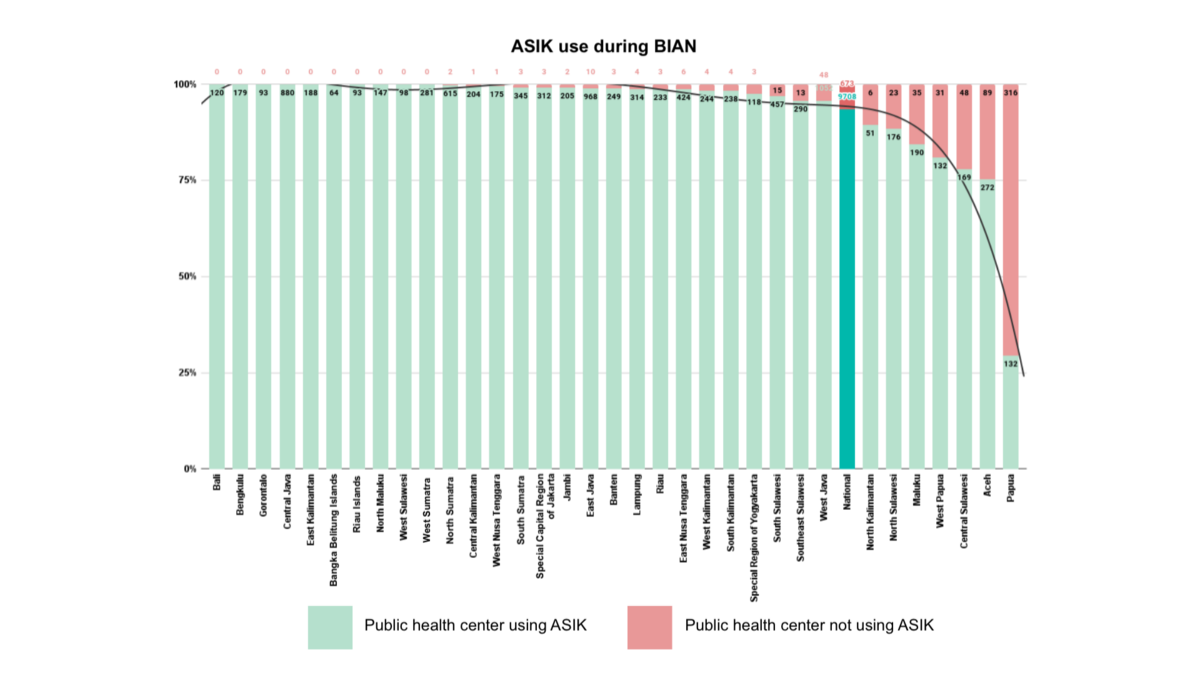
Aplikasi Sehat IndonesiaKu (ASIK) use across 34 provinces during the Bulan Imunisasi Anak Nasional (BIAN) implementation.

To understand the aforementioned discrepancies, 2 further aspects were considered: ASIK use and coverage and user feedback on ASIK use. The results will be presented in the following paragraphs. Regarding ASIK coverage, a total of 93.5% (9708/10,382) of public health centers, 93.5% (6478/6928) of subdistricts, and 97.5% (501/514) of districts and cities in 34 provinces reported BIAN activities using ASIK. On the basis of the coverage as measured at the province level, there were 10 provinces where 100% of their public health centers reported BIAN activities using ASIK, namely, Bali, Bengkulu, Gorontalo, Central Java, East Kalimantan, Bangka Belitung Islands, Riau Islands, North Maluku, West Sulawesi, and West Sumatra, whereas the provinces with the lowest number of public health centers reporting using ASIK were Papua (132/448, 29.5%), Aceh (272/361, 75.3%), and Central Sulawesi (169/217, 77.9%). Detailed information on other provinces can be found in Figure 4.

### User Feedback on ASIK Use

In parallel to understanding the coverage and use of ASIK, a quantitative survey was conducted to obtain users’ feedback on the system, including explorations of UI and UX, reporting variables, and system interference. A total of 1065 questionnaires were received, although not all respondents answered all questions. The results of the quantitative survey are presented in this section and summarized in Table 1. In general, users exhibited a good understanding of ASIK as the EIR (650/809, 80.3%). For UI and UX, the highest feedback was that it was overall good but could still be improved (489/793, 61.7%), with 35.3% (280/793) of respondents expressing that the UI and UX were very good and 3% (24/793) saying that they were hard to understand. Regarding the required variables for immunization data input in ASIK, 54% (422/781) of the respondents expressed that the variables fit their needs but could still be improved, 39.6% (309/781) expressed that the variables fit their needs, and 6.4% (50/781) of users expressed that there were too many variables. In terms of system interferences, 59.1% (463/784) of users expressed that they sometimes happened, and 24.5% (192/784) expressed that they often happened. The most prevalent system interferences were (1) inability to find individual data in the system (281/779, 36.1%), (2) server errors (217/779, 27.9%), and (3) problems with logistic stock confirmation (135/779, 17.3%).

On the basis of the users’ feedback, 44% (382/869) stated that there were 2 to 3 staff members that helped with the data reporting process. However, the number of staff members who helped with data input varied as users in region 2 (high data collection) had >5 staff members for data reporting and users in region 1 (low data collection) only had 2 to 3 staff members to do so. Regarding the flow of data reporting, 33.4% (291/872) of users exhibited a combination of recording the immunization data on paper or on Microsoft Excel first and directly inputting them into ASIK. Furthermore, more users preferred the input of immunization data at public health centers right after the immunization activity was completed (364/1089, 33.4%), followed by inputting the data within 24 hours after the immunization services (203/1089, 18.6%). However, 22.3% (181/813) of users in region 1 input the immunization data 2 weeks after the immunization activity, whereas in region 2, a total of 45.3% (125/276) of their users preferred to input the data right after the activity was completed. Regarding the data reporting process, each region had different characteristics for each variable: number of staff members, data reporting flow, and time to input the data. Region 2 showed a relatively higher number of staff members, more direct flow of data reporting, and timelier input of data into the system. As previously mentioned, region 2 had the highest data collection, comprising 28 cities that did not have any gaps in data collection between ASIK and manual reporting.

**Table 1:** Quantitative exploration of the use of Aplikasi Sehat IndonesiaKu (ASIK; N=1065).

Characteristic				Region 1/ (n=800), n/ (%)		Region 2/ (n=265), n/ (%)		Total, n/ (%)
**Overall user feedback on the system**								
	**System understanding**							
		Limited understanding	135/598 (22.6)		24/211 (11.4)		159/809 (19.7)	
		Good understanding	463/598 (77.4)		187/211 (88.6)		650/809 (80.3)	
	**User interface and user experience**							
		Very good	200/587 (34.1)		80/206 (38.8)		280/793 (35.3)	
		Overall good but can still be improved	366/587 (62.4)		123/206 (59.7)		489/793 (61.7)	
		Hard to understand	21/587 (3.6)		3/206 (1.5)		24/793 (3)	
	**Variables**							
		Fit with needs	213/576 (37)		96/205 (46.8)		309/781 (39.6)	
		Fit with needs but can still be improved	321/576 (55.7)		101/205 (49.3)		422/781 (54)	
		Too many variables	42/576 (7.3)		8/205 (3.9)		50/781 (6.4)	
	**System interference**							
		Seldom	18/579 (3.1)		3/205 (1.5)		21/784 (2.7)	
		Rarely happened	58/579 (10)		36/205 (17.6)		94/784 (12)	
		Sometimes happened	326/579 (56.3)		137/205 (66.8)		463/784 (59.1)	
		Often happened	163/579 (28.2)		29/205 (14.1)		192/784 (24.5)	
		Always happened	14/579 (2.4)		0/205 (0)		14/784 (1.8)	
**Data reporting process**								
	**Number of staff members for data input**							
		1 person	95/645 (14.7)		8/224 (3.6)		103/869 (11.9)	
		2-3 people	311/645 (48.2)		71/224 (31.7)		382/869 (44)	
		4-5 people	98/645 (15.2)		43/224 (19.2)		141/869 (16.2)	
		>5 people	141/645 (21.9)		102/224 (45.5)		243/869 (28)	
	**Flow of data reporting**							
		(1) Recording the data on paper or manual book report before inputting them into ASIK	153/647 (23.6)		35/225 (15.6)		188/872 (21.6)	
		(2) Recording the data in Microsoft Excel sheet before inputting them into ASIK	119/647 (18.4)		20/225 (8.9)		139/872 (15.9)	
		(3) Directly inputting the data into ASIK	51/647 (7.9)		46/225 (20.4)		97/872 (11.1)	
		Combination of 1 and 2	131/647 (20.2)		26/225 (11.6)		157/872 (18)	
		Combination of 1, 2, and 3	193/647 (29.8)		98/225 (43.6)		291/872 (33.4)	
	**Time to input data into ASIK**							
		Directly at the vaccination center	78/813 (9.6)		60/276 (21.7)		138/1089 (12.7)	
		Directly after the activity at the public health centers	239/813 (29.4)		125/276 (45.3)		364/1089 (33.4)	
		Input the data within a day at home	161/813 (19.8)		42/276 (15.2)		203/1089 (18.6)	
		Input the data within 3 days of manual data collection	86/813 (10.6)		14/276 (5.1)		100/1089 (9.2)	
		Input the data within 7 days of manual data collection	68/813 (8.4)		14/276 (5.1)		82/1089 (7.5)	
		Input the data ≥2 weeks after manual data collection	181/813 (22.3)		21/276 (7.6)		202/1089 (18.5)	
**Existing infrastructure**								
	**Android mobile phone ownership**							
		Yes	694/711 (97.6)		229/241 (95)		923/952 (97)^y^	
		No	17/711 (2.4)		12/241 (5)		29/952 (3)^y^	
	**Internet access**							
		Available at all places	390/710 (54.9)		163/240 (67.9)		553/950 (58.2)	
		Only available at the public health centers	78/710 (11)		14/240 (5.8)		92/950 (9.7)	
		Limited availability	242/710 (34.1)		63/240 (26.3)		305/950 (32.1)	
**Data completeness**								
	**Data availability**							
		Individual data were complete	157/637 (24.6)		125/219 (57.1)		282/856 (32.9)	
		Individual data were incomplete	480/637 (75.4)		94/219 (42.9)		574/856 (67.1)	

Regarding the existing infrastructure, 97% (923/952) of users had Android mobile phones. A total of 58.2% (553/950) of respondents expressed that internet access was available at all places, 32.1% (305/950) expressed that internet availability was limited in several places, and 9.7% (92/950) expressed that the internet was only available at public health centers (and not available in immunization posts). The data suggested that the internet was more accessible in region 2 compared to region 1.

Another constraint found during data reporting was data completeness of the targeted participants. A total of 67.1% (574/856) of users expressed that the individual data were incomplete, especially on unique identifier (ID number), which was only available for up to 79% of the collected data points; full name, which was available for up to 95% of individual data points; birth date, which was available for up to 95% of the collected data points; and gender, which was available for up to 95% of the collected data points.

Most respondents (618/1065, 58%) indicated several constraints that hindered the use of ASIK and were experienced intermittently or too frequently, including (1) challenges in retrieving individual data, (2) server errors, and (3) logistic stock validation. The first challenge that arose in the early development of the system is the lack of interconnectedness with the civil registration database. During the preliminary development phase of ASIK, the absence of established data sharing agreements between the Indonesian MoH and the Ministry of Home Affairs prevented users from efficiently engaging in data reporting using ASIK.

Interconnection between ASIK and the civil registration database will help health care workers automatically retrieve individual demographic information such as gender, date of birth, and address by inputting the 16 digits of the national ID. During BIAN implementation, ASIK was still not connected to the civil registration database owned by the Ministry of Home Affairs; thus, health care workers needed to input the individual information manually.

The second challenge also stemmed from the early development phase of the system, when the initial server capacity was constrained, leading to suboptimal application performance. System errors that were mentioned by the respondents happened occasionally throughout the recording process. They sometimes happened due to personal data input, vaccine type data input, or the process of saving data. They potentially happened due to limited server performance caused by the high number of users inputting data at the same time.

The third challenge pertains to logistic stock management. In contrast to the first challenge, ASIK was strategically integrated with the logistic system (SMILE) during its development phase, effectively establishing a linkage between immunization and logistic data. However, the compliance and adherence to using the SMILE were variable. Consequently, the comprehensive implementation of the SMILE for monitoring logistic stocks in the context of the BIAN 2022 campaign was not universal across all public health centers. This posed an obstacle in the use of ASIK given that logistic confirmation is a prerequisite for inputting immunization data into ASIK. Respondents were hindered from inputting the immunization data into ASIK if the pharmacy staff had not input the logistic information into the SMILE. This created some delays in the data recording process during BIAN 2022.

These challenges have been addressed, and the system has been enhanced over time, particularly during the latter stage of the BIAN 2022 campaign. The system was integrated with the civil registration database, the server capacity was upgraded, and refinements were implemented in the integration between ASIK and the SMILE. As a result, the efficacy of ASIK had a notable improvement during the latter phase of the BIAN 2022 campaign. Nevertheless, many respondents gave positive responses for the use of ASIK as the Indonesia EIR. The positive response correlated with the convenience of ASIK as an EIR.

## Discussion

### Principal Findings

The launch of ASIK is a part of the MoH’s mission to transform health care services by assisting health care workers in efficiently and comprehensively recording patient data in a single, integrated database [5,15,19]. The use of ASIK as Indonesia’s first EIR was broad, covering 34 out of 38 provinces. The rate of adoption reached 93.5% (9708/10,382) of public health centers (ie, the main health care provider for the nationwide immunization campaign). Through 2 phased releases, ASIK managed to record >21 million immunizations. Although most users expressed a good understanding of the system, several challenges persisted during the implementation phase.

EIRs are instruments proven to be cost-effective in enhancing coverage and timeliness of vaccination [23]. The use of EIRs was considered beneficial in preventing delays in vaccinating children as they help parents keep track of their children’s vaccination records and stay informed of their children’s vaccination schedule [23,24]. Furthermore, EIRs are helpful to identify high-risk populations; facilitate resource and activity planning, such as monitoring vaccination coverage and vaccine stock availability; and determine the overall performance of the immunization program [23,24]. During ASIK’s first launch, Indonesia’s MoH emphasized that, in the future, children who have received immunization will have their records digitally stored in ASIK, in which the integrated data aim to facilitate parents’ access to their children’s immunization data, especially to be used for school-related purposes [19].

In the technical implementation guideline document for the BIAN, recording and reporting of immunization activities are mandated to be conducted electronically through ASIK [7]. Despite electronic data input being mandatory, the data recorded in ASIK did not represent the complete immunization service during the BIAN 2022 campaign. According to the ASIK analytical dashboard, the national coverage recorded in ASIK for MR, OPV, IPV, and DPT-HB-Hib immunization was 50.1% (18,301,057/36,497,694), 36.2% (938,623/2,595,240), 30.7% (1,276,668/4,158,289), and 40.2% (1,371,104/3,407,900), respectively. In addition, there was an average discrepancy of approximately 19% between digital and manual data reporting. When compared to similar supplementary immunization efforts in previous years, the Polio National Immunization Week held in 2016 achieved an impressive coverage rate of 96.5%. MR supplementary immunization activities in 2017 exceeded expectations, reaching >100.9%, whereas in 2018, the coverage stood at approximately 73.4% [7]. Around the same time, between 2017 and 2019, several countries, including Afghanistan, Benin, Ghana, Timor-Leste, Togo, Sierra Leone, Senegal, Rwanda, Pakistan, Niger, Malawi, Lesotho, and Ethiopia, also attained MR supplementary activity coverage rates of >90% [25]. Nonetheless, it is important to consider that these coverage data were obtained from manual or paper-based immunization records and postcampaign surveys [25].

Considering that the immunization data are stored in different files segregated by health center and area, it is nearly impossible to ensure that there is no duplication of data in each record. Studies show that paper-based data reports actually create challenges such as affecting health care workers’ ability to make data-driven decisions by analyzing the manual immunization reports and increasing the burden of health workers to input data both in the paper-based and digital reporting systems [26]. Furthermore, the use of paper-based aggregate data for analysis presents gaps, such as limited individual longitudinal data, and omits crucial sociodemographic information [27]. Data stored electronically enable prompt data retrieval, hence facilitating immediate reports related to disease and surveillance, which is in contrast to paper-based reports that rely on manual retrieval [13]. Dual use of EIR and paper-based immunization reports further exacerbates the challenges of data input using ASIK for health care workers. A transition from paper-based reports to paperless reports and dual entry processes are also perceived barriers and challenges to EIR implementation in other countries such as Kenya and Vietnam [26,28]. The immunization program has grown in complexity and leads to an increased need for data, and the immunization information system must be flexible to cater to those needs [13]. Paper-based reports are limited in meeting such needs as they are heavily influenced by population mobility and errors stemming from estimation of population number [13]. Hence, moving forward, it is important for relevant stakeholders to decide whether to solely use paper-based reports or onboard using ASIK for Indonesia immunization programs.

On the other hand, data captured in ASIK represent individual data, which can be linked to personal immunization history records. Individual immunization data recorded in an EIR such as ASIK offer the advantage of providing precise and timely information. This, in turn, facilitates critical activities such as monitoring vaccination compliance, mitigating dosing errors, and identifying unvaccinated individuals [29]. The significance of individual immunization records in clinical decision-making is noteworthy as they provide a comprehensive historical vaccination record and diminish the likelihood of misclassifying vaccination status [30]. Consequently, this aids in reducing instances of both under- and overimmunization in children [31]. Furthermore, integrating individual immunization data from an immunization information system into an electronic medical record or electronic health record can enhance the accuracy of vaccine safety assessments [30,32].

ASIK has achieved significant engagement, involving 93.5% (9708/10,382) of public health centers across Indonesia within a span of 7 months since its launch. This indicates that public health centers were informed about the data input process and user guide updates that come with it, although long-term adherence to data input remains to be seen. The high level of ASIK use might be supported by several aspects, such as the compatibility of the Android app in Indonesia, the user-friendliness of the system interface, and the ease of use. Considering the high level of mobile phone and internet use in Indonesia, the digitization of health service reports is happening apace [33]. In 2021, approximately 90.5% of households in Indonesia owned at least one mobile phone, covering 65.8% of the population, and approximately 62% of the population had access to the internet [34]. Indonesia is estimated to have >200 million mobile phone users and is dominated by Android operating systems (95%) [34,35]. This is in line with our findings, in which 97% (923/952) of respondents stated that they owned Android-based mobile devices. ASIK is built on an Android operating system, which is compatible with various mobile devices, allowing health care workers in Indonesia to benefit from it [33]. Existing EIRs such as ImmunizaCA in Canada, Zindagi Mehfooz in Pakistan, and DHIS2 Android Capture App in Zambia are also built on Android-based platforms [36-40].

A visually appealing or user-friendly UI and UX can prompt users to explore an app, facilitating a comprehensive understanding of its available functions, consequently influencing user acceptability [41,42]. It is essential to acknowledge that ASIK signifies a transition for health care workers from paper-based to electronic reporting; hence, data and variables should be both useful and recognizable to the health workers as it is important to note that a familiar interface correlates with use frequency [43]. Other than that, a familiarity with workflow and a design that carefully considers health care workers’ needs are crucial for effective EIR implementation [26].

Despite the users’ acceptability of ASIK as an EIR, the low number of reporting in certain provinces may be due to several factors. First, primary health care workers, often responsible for numerous data collection tasks, are frequently burdened with heavy workloads, lack motivation, and view data recording as undesirable and time-consuming [44,45]. Such excessive workloads can compromise the quality of health care data reported at health facilities, leading to inaccuracies and unreliability when other tasks interfere with data entry [46,47]. Indeed, the lowest compliance was often correlated with the provinces that have the lowest ratios of primary health care staff, and thus, it is presumed that staff time availability is the strongest root cause for the observed variation in ASIK use. The workload also affects workers’ opportunities to adequately familiarize themselves (digital familiarization) with any health information system they are about to use, often coupled with inadequate handover and shortage of available staff for this knowledge transfer [48,49]. In the next phase, to ensure that there is an appropriate and sustainable knowledge transfer to all end users, conducting a cascading training-of-trainers approach and establishing a multilevel technical support network is recommended [28].

The sudden explosion in the number of health-related apps in Indonesia, with >400 health-related apps developed and introduced for health care facilities, further exacerbates the burden on health workers regarding digital familiarization and data input [15]. In addition, lack of adequate monitoring by supervisors or managers and provision of technical support affects the effective implementation of data reports through an information system [48,49]. Furthermore, the BIAN implementation coincided with a massive COVID-19 preventive immunization effort, adding more tasks to primary care workers’ daily workload [50,51]. The implementation of COVID-19 immunization entailed a collaborative approach involving various sectors, with health personnel sourced from governmental, military, and private practitioner sectors [52]. This stands in stark contrast to the BIAN 2022 campaign, which relied heavily on health workers stationed at public health center facilities [52]. Therefore, there was a double burden for primary care workers to reach both the target for COVID-19 immunization and the target for the BIAN 2022 campaign and report both results using different digital pathways. This further justified the need to simplify, integrate, and ensure the interoperability of the health information system in Indonesia, as mentioned in the digital transformation blueprint, and ensure that the EIR is developed to match users’ needs and able to simplify the reporting workflow accordingly [15,26].

In the process of implementing digital technologies in the health care sector, certain challenges are inevitable [53]. These challenges encompass a range of factors, including but not limited to client identification and verification as well as lack of database integration [53,54]. In ASIK, the collection of the national identification number of the service recipient is a prerequisite for generating an immunization service record. Indonesia uses a 16-digit identification number, known as the resident registration number, which serves as a cornerstone of national demographic identification [55]. Current data indicate that 97.2% of Indonesian citizens already possess a unique identification number, but there are also 5.38 million individuals without unique identification numbers [55,56]. During BIAN implementation, there were many instances in which patients did not bring any personal identification tools when immunization services were provided. Consequently, users were burdened to create individual data without unique identifiers while also needing to remember that exact record for identification and retrieval in the future when the same patient visited. Lack of linkage and integration with other databases were also a common challenge, which happened in the development of EIRs in Africa [54]. It is crucial to foster awareness and establish a mutual understanding between the realms of public health and IT to facilitate EIR development [54]. Ensuring that EIRs comprehensively covered all regions across Indonesia, encompassing an estimated target population of 37 million individuals for BIAN immunization, presented a challenge in conveying this requirement effectively, especially in the beginning of the system’s development. To build an interoperable EIR system, integration with the national ID database is crucial [28]. Moving forward, the MoH could establish a continuous collaboration with the Ministry of Home Affairs as the primary stakeholders and foster understanding of and commitment to the need for database integration and providing immediate legal identity to the population since birth.

To report immunization data in ASIK, having access to the internet is a prerequisite. Despite statistics indicating that approximately 62% of Indonesia’s population use the internet and the country’s consistent progress in improving internet connectivity, disparities persist in terms of access across different social classes and regions [34,57]. Notably, significant portions of Indonesia, particularly in the eastern regions, still lack access to the internet [58]. The distribution of infrastructure for signal reception in Indonesia reveals that >3023 villages lack signal reception infrastructure, with >934 of these villages located in Papua [34]. Given the limited internet access, it is unsurprising that the coverage of MR, OPV, IPV, and DPT-HB-Hib immunization reports using ASIK remained low in Papua. Mobile apps that need to constantly rely on internet connectivity reduce effectiveness for users such as health care workers that operate in remote regions [59]. To reduce the digital divide among regions, the government could explore the potential use of satellite-based cellular and internet connectivity for rural areas [60]. This could be done through collaboration with the Ministry of Communication and Information Technology or alongside existing internet provider entities. Other than ensuring internet connectivity, support and assistance to infrastructure and human resources is paramount, particularly in areas with low information and communications technology maturity scores [61].

This is the first study that describes the EIR in Indonesia, which replaces the manual reporting system from the MoH, including describing its use during the BIAN. Furthermore, the quantitative survey provided an overview of the obstacles in the implementation of ASIK for the BIAN program and provided input on the features that need to be improved for future immunization data collection processes. However, a relative weighing of the factors that require future improvement was not sought and, thus, can become the subject of future research.

This study has several limitations that need to be considered. First, ASIK is only available on Android-based platforms and has not been released for iOS; thus, data reporting is limited to officers who own devices compatible with Android. Second, the quantitative survey that was administered covered a limited number of the actual ASIK users in the field, but it is expected that this number can represent feedback from the field. In addition, using Mentimeter potentially limited the feedback from some of the participants when they did not fill the questionnaire in a timely manner, and considering that the evaluation meeting was conducted on the web, there was a risk of limited control over the participants filling out the questionnaire in a timely manner. Finally, the manual data that were compared to ASIK are the result of a report from the provincial health office, which might not fully capture the recording process that took place. Nevertheless, this study provides a deeper understanding of the use of ASIK in the BIAN program as the first EIR implemented nationally by the MoH.

### Conclusions

EIRs have demonstrated their cost-effectiveness in improving vaccination coverage and timeliness, offering numerous advantages in disease prevention and health care management. Adoption and continuous use of EIRs entail numerous challenges, especially in a country such as Indonesia with an enormous number of targeted immunization populations and limited infrastructure settings. Challenges observed from this study included the gap between manually reported data and electronic records due to dual data entry processes; heavy workloads for primary care health workers to carry out immunization campaigns as well as the reporting process; lack of data completeness in ID number, full name, date of birth, or gender; system interferences due to the early development phase of the system; and limited infrastructure settings to support digital connection in remote areas. Addressing these challenges and ensuring adequate support for health care workers are essential steps in enhancing the effectiveness of EIRs in improving public health outcomes.

Although faced with the aforementioned challenges, ASIK as Indonesia’s first national EIR managed to reach substantial engagement. More than 93% of its targeted users adopted the system, and this was potentially made possible by ease of system use, continuous system improvement according to users’ feedback, and immediate regulation support. Ease of use and user-friendly apps that simplify data input processes are essential to reinforce user acceptability. In the future, ASIK can be developed for a wider immunization program provided that improvement of features is carried out, including the preparation of relevant human resources and supporting digital infrastructure and stronger regulatory support.

## Appendix

Multimedia Appendix 1 Questionnaire.

Multimedia Appendix 2 Bulan Imunisasi Anak Nasional 2022 campaign target.

Multimedia Appendix 3 Comparison of measles-rubella vaccine; oral polio vaccine; inactivated polio vaccine; and diphtheria, pertussis, tetanus, hepatitis B, and Haemophilus influenzae type B immunization coverage using Aplikasi Sehat IndonesiaKu data and manual report data.

Multimedia Appendix 4 Detail of measles-rubella vaccine; oral polio vaccine; inactivated polio vaccine; and diphtheria, pertussis, tetanus, hepatitis B, and Haemophilus influenzae type immunization coverage using Aplikasi Sehat IndonesiaKuASIK data and manual report data.

## Abbreviations

ASIK: Aplikasi Sehat IndonesiaKu
BIAN: Bulan Imunisasi Anak Nasional
DPT-HB-Hib: diphtheria, pertussis, tetanus, hepatitis B, and Haemophilus influenzae type B
EIR: electronic immunization registry
IPV: inactivated polio vaccine
MoH: Ministry of Health
MR: measles-rubella
OPV: oral polio vaccine
SMILE: Electronic Logistic Management and Information System
UI: user interface
UX: user experience

## Data Availability

The datasets generated or analyzed during this study are available from the corresponding author on reasonable request.
