# Supplementary material for: Using an Electronic Immunization Registry (Aplikasi Sehat IndonesiaKu) in Indonesia: Cross-Sectional Study
Source: Interact J Med Res. 2025 Mar 27;14:e53849. doi: 10.2196/53849 (PMC11986391; doi:10.2196/53849)
Supplement: Multimedia Appendix 1 [file ijmr_v14i1e53849_app1.docx]

# Multimedia Appendix 3: Questionnaire

#

# Questionnaire Guidelines

**Interview objectives:**

Identifying availability, challenges, strengths, weaknesses, and opportunities for improvement (including systems, infrastructure, data completeness) in the use of Aplikasi Sehat IndonesiaKu (ASIK) in immunization data recording.

Thank you for agreeing to participate in this interview - we really appreciate your time. This activity is part of the efforts of the Data and Information Center - Ministry of Health, through the Digital Transformation Office (DTO) to explore various information related to Aplikasi Sehat IndonesiaKu (ASIK) in immunization data recording.

| **Question** |
| --- |
| 1. Opening 2. Which Province do you come from? 3. Which District/city do you come from? 4. Which Institution do you come from? 5. What is your role in BIAN activity? 6. What is your position in the Health Service? |
| **Quantitative Assessment of Aplikasi Sehat IndonesiaKu for immunization data recording**   1. Interview with key points:    1. Overall feedback on system    2. Data reporting process    3. Infrastructure    4. Data completeness |

# Questionnaire

| *Part 1: Overall feedback on system* | |
| --- | --- |
| *Question* | *Answer* |
| 1. *How do you understand the system?*   Guide to asking:  *We would like to know about your general understanding about the system. Do you have limited understanding or good understanding?* | 1. Do not understand everything 2. A little understanding 3. Quite understanding 4. Very understanding   (Probing: How familiar are you with the system? How do you use the system in your daily tasks?) |
| 1. *How intuitive do you find the system’s interface?*   Guide to asking:  *We would like to know if the system interface (UI/UX) has intuitive looks to be used* | 1. Very good 2. Overall good but still can be improve 3. Hard to understand   (Probing: Do you think all the system interference is easy to use? Or are there some of the system interferences that are hard to understand?) |
| 1. *Are the system's variables enough to meet your needs?*   Guide to asking questions  *We want to know whether the system’s variables are enough to meet the user's needs.* | 1. Fit with the needs 2. Fit with the needs but still can be improve 3. Too many variable   (Probing: Are the system's variables flexible enough to adapt to your specific needs and tasks? If not, what aspects do you think could be improved to make it more suitable for your requirements?) |
| 1. *Have you encountered any challenges or issues while using the system?*   Guide to asking questions  *We want to know whether the system’s interference.* | 1. Seldom 2. Rarely happened 3. Sometimes happened 4. Often happened 5. Always happened   (Probing: Have you encountered any challenges or issues while using the system? If so, could you please describe the specific challenges and how they affected your work?) |

| *Part 2: Data Reporting Process* | |
| --- | --- |
| *Question* | *Answer* |
| 1. *How many staff members are typically involved in the data input process for the system?*   Guide to asking:  *We would like to know about the number of staff who responsible for data input process for the system.* | 1. 1 person 2. 2-3 people 3. 4-5 people 4. >5 people   (Probing: How many staff members are typically involved in the data input process for the system? Please provide an approximate number. Do all staff members participate equally in the data input process, or do certain team members have more involvement than others?) |
| 1. *Can you describe the steps involved in the data reporting process, from data collection to final input into the system?*   Guide to asking:  *We would like to know about data reporting flow regarding the system.* | 1. Record the data on paper/manual book before input the data into the ASIK 2. Record the data into excel sheet before input the data into the ASIK 3. Directly input the data into the ASIK 4. Combination I & II 5. Combination I, II, & III   (Probing: How is the data collected initially, and what tools or methods are used? Who is responsible for each step in the data reporting process?) |
| 1. *When do you or your team input data into the system?*   Guide to asking questions  *We want to know the time to input data to the system in Puskesmas.* | 1. Direct in vaccination centre 2. Direct after activity in Puskesmas 3. Input the data within a day in home 4. Input after three days collecting manual data 5. Input after seven days collecting manual data 6. Input after two weeks or more collecting manual data   (Probing: Is data input done at regular intervals (daily, weekly, monthly), or does it depend on specific events or triggers? Do you find that the current schedule for data input works well for your team, or would you prefer a different timing or frequency?) |

| *Part 3: Existing Infrastructure* | |
| --- | --- |
| *Question* | *Answer* |
| 1. *Do you own an Android mobile phone?*   Guide to asking:  *We would like to know about the number of staff who own android mobile phones.* | 1. Yes 2. No   (Probing: If yes, what model of Android phone do you own? If you don’t own an Android phone, what type of mobile phone do you use instead?) |
| 1. *Do you have access to the internet in your area?*   Guide to asking:  *We would like to know about internet access in your area.* | 1. Available at all places 2. Only available at Puskesmas 3. Limited availability   (Probing: Is high-speed internet available in your area, and do you have access to it? How reliable is your internet connection? Do you experience frequent outages or interruptions?) |

| *Part 4: Data Completeness* | |
| --- | --- |
| *Question* | *Answer* |
| 1. *How complete is the data available for input into the system?*   Guide to asking:  *We would like to know about the completeness of individual data to be inputted into the system, e.g. id number, full name, date of birth, sex, and parents information.* | 1. Individual data are complete 2. Individual data are incomplete   (Probing: If incomplete, what types of data are missing or lacking? How would you rate the quality of the data available for input into the system?) |
