# Supplementary material for: Using an Electronic Immunization Registry (Aplikasi Sehat IndonesiaKu) in Indonesia: Cross-Sectional Study
Source: Interact J Med Res. 2025 Mar 27;14:e53849. doi: 10.2196/53849 (PMC11986391; doi:10.2196/53849)
Supplement: Multimedia Appendix 2 [file ijmr_v14i1e53849_app2.docx]

### Multimedia Appendix

Appendix 1 BIAN 2022 Campaign Target

| **Province** | **Target** | |
| --- | --- | --- |
|  | **Measles-rubella** | **OPV, IPV, DPT-HB-Hib** |
| Aceh | 95% | 80% |
| Bali* | - | 80% |
| Banten | 95% | 80% |
| Bengkulu | 95% | 80% |
| DKI Jakarta | 95% | 80% |
| Special Region of Yogyakarta* | - | 80% |
| Gorontalo | 95% | 80% |
| Jambi | 95% | 80% |
| West Java | 95% | 80% |
| Central Java | 95% | 80% |
| East Java | 95% | 80% |
| West Kalimantan | 95% | 80% |
| South Kalimantan | 95% | 80% |
| Central Kalimantan | 95% | 80% |
| East Kalimantan | 95% | 80% |
| North Kalimantan | 95% | 80% |
| Bangka Belitung Islands | 95% | 80% |
| Riau islands | 95% | 80% |
| Lampung | 95% | 80% |
| Maluku | 95% | 80% |
| North Maluku | 95% | 80% |
| West Nusa Tenggara | 95% | 80% |
| East Nusa Tenggara | 95% | 80% |
| Papua | 95% | 80% |
| West Papua | 95% | 80% |
| Riau | 95% | 80% |
| West Sulawesi | 95% | 80% |
| South Sulawesi | 95% | 80% |
| Central Sulawesi | 95% | 80% |
| Southeast Sulawesi | 95% | 80% |
| North Sulawesi | 95% | 80% |
| West Sumatra | 95% | 80% |
| South Sumatra | 95% | 80% |
| North Sumatra | 95% | 80% |
