# Supplementary material for: Using an Electronic Immunization Registry (Aplikasi Sehat IndonesiaKu) in Indonesia: Cross-Sectional Study
Source: Interact J Med Res. 2025 Mar 27;14:e53849. doi: 10.2196/53849 (PMC11986391; doi:10.2196/53849)
Supplement: Multimedia Appendix 3 [file ijmr_v14i1e53849_app3.docx]

Appendix 2 Comparison of MR, OPV, IPV, and DPT-HB-Hib Immunization coverage using ASIK data and manual report

| **Province** | **Immunization Coverage (%)** | | | | | | | | | | | |
| --- | --- | --- | --- | --- | --- | --- | --- | --- | --- | --- | --- | --- |
|  | **MR** | | | **OPV** | | | **IPV** | | | **DPT-HB-Hib** | | |
|  | **Manual report (%)** | **ASIK (%)** | **Gap (%)** | **Manual report (%)** | **ASIK (%)** | **Gap (%)** | **Manual report (%)** | **ASIK (%)** | **Gap (%)** | **Manual report (%)** | **ASIK (%)** | **Gap (%)** |
| Aceh | 19.4 | 6.45 | 12.95 | 36.7 | 14.87 | 21.83 | 6.2 | 2.7 | 3.5 | 13 | 3.95 | 9.05 |
| Bali* | N/A | N/A | N/A | 107.4 | 84.7 | 22.7 | 106.3 | 87.94 | 18.36 | 99.1 | 103.12 | -4.02 |
| Bangka Belitung Islands | 60.5 | 35.94 | 24.56 | 28.2 | 15.71 | 12.49 | 14.2 | 5.16 | 9.04 | 46.3 | 19.07 | 27.23 |
| Banten | 98.6 | 96.75 | 1.85 | 102.4 | 105.18 | -2.78 | 97.5 | 94.31 | 3.19 | 97.2 | 109.38 | -12.18 |
| Bengkulu | 69.2 | 39.68 | 29.52 | 20 | 21.76 | -1.76 | 18.4 | 15.76 | 2.64 | 29.9 | 20.74 | 9.16 |
| Central Java | 97.9 | 96.45 | 1.45 | 106.7 | 87.89 | 18.81 | 97.9 | 86.51 | 11.39 | 98.6 | 85.15 | 13.45 |
| Central Kalimantan | 64.9 | 40.63 | 24.27 | 9.4 | 4.89 | 4.51 | 4.6 | 1.99 | 2.61 | 13.5 | 4.86 | 8.64 |
| Central Sulawesi | 57.1 | 14.72 | 42.38 | 30.9 | 7.25 | 23.65 | 15.2 | 2.04 | 13.16 | 25.8 | 4.67 | 21.13 |
| Jakarta | 99.3 | 74.67 | 24.63 | 19.8 | 21.69 | -1.89 | 9.3 | 11.11 | -1.81 | 74.8 | 77.44 | -2.60 |
| East Java | 100.6 | 76.4 | 24.2 | 97.8 | 27.38 | 70.42 | 91.9 | 21.15 | 70.75 | 92.2 | 20.64 | 71.56 |
| East Kalimantan | 70.3 | 45.05 | 25.25 | 10.1 | 2.5 | 7.6 | 4.1 | 1.11 | 2.99 | 23.5 | 5.34 | 18.16 |
| East Nusa Tenggara | 66.6 | 50.25 | 16.35 | 24.6 | 19.05 | 5.55 | 23.9 | 21.32 | 2.58 | 35.5 | 30.88 | 4.62 |
| Gorontalo | 56.9 | 38.47 | 18.43 | 71.7 | 11.06 | 60.64 | 23.5 | 3.43 | 20.07 | 22.7 | 15 | 7.7 |
| Jambi | 81.1 | 38.82 | 42.28 | 69.4 | 31.05 | 38.35 | 66.5 | 20.6 | 45.9 | 77.6 | 25.3 | 52.3 |
| Lampung | 82 | 67.35 | 14.65 | 92 | 131.74 | -39.74 | 85.5 | 74.31 | 11.19 | 89.2 | 89.57 | -0.37 |
| Maluku | 69.3 | 21.94 | 47.36 | 61.5 | 11.17 | 50.33 | 17.6 | 3.69 | 13.91 | 73.5 | 15.35 | 58.15 |
| North Kalimantan | 61.1 | 36.46 | 24.64 | 17.9 | 5.07 | 12.83 | 12.6 | 4.25 | 8.35 | 30.1 | 10.54 | 19.56 |
| North Maluku | 68.8 | 38.95 | 29.85 | 26.3 | 18.76 | 7.54 | 26.2 | 11.55 | 14.65 | 26.1 | 13.64 | 12.46 |
| North Sulawesi | 61.2 | 25.1 | 36.1 | 28.5 | 8.66 | 19.84 | 14.6 | 3.08 | 11.52 | 50.5 | 11.97 | 38.53 |
| North Sumatra | 73.4 | 49.1 | 24.3 | 75.2 | 61.36 | 13.84 | 55 | 38.18 | 16.82 | 61.4 | 51.64 | 9.76 |
| Papua | 38.8 | 9.13 | 29.67 | 11.6 | 0.8 | 10.8 | 3.3 | 1.08 | 2.22 | 5 | 0.67 | 4.33 |
| Riau | 46 | 29.33 | 16.67 | 14.5 | 5.62 | 8.88 | 7.1 | 3.88 | 3.22 | 14.8 | 4.99 | 9.81 |
| Riau Islands | 71.6 | 42.6 | 29 | 81.5 | 27.87 | 53.63 | 64.5 | 8.02 | 56.48 | 91.5 | 9.1 | 82.4 |
| South Kalimantan | 54.1 | 42.49 | 11.61 | 29.1 | 22.95 | 6.15 | 14.2 | 11.22 | 2.98 | 31.5 | 24.45 | 7.05 |
| South Sulawesi | 95.3 | 48.01 | 47.29 | 43.6 | 19.17 | 24.43 | 31 | 14.05 | 16.95 | 29 | 11.24 | 17.76 |
| South Sumatra | 68.3 | 46.82 | 21.48 | 84 | 61.36 | 22.64 | 57.3 | 33.78 | 23.52 | 99.2 | 68.21 | 30.99 |
| Southeast Sulawesi | 52.1 | 26.81 | 25.29 | 14.9 | 7.3 | 7.6 | 8.3 | 4.17 | 4.13 | 21.2 | 6.37 | 14.83 |
| Special Region of Yogyakarta* | N/A | N/A | N/A | N/A | N/A | N/A | 92.2 | 14.78 | 77.42 | 96.7 | 52.38 | 44.32 |
| West Java | 95.7 | 68.78 | 26.92 | 96.1 | 59.17 | 36.93 | 92 | 57.52 | 34.48 | 89.4 | 53.62 | 35.78 |
| West Kalimantan | 58.5 | 39.31 | 19.19 | 8.4 | 5.06 | 3.34 | 10.7 | 6.2 | 4.5 | 10.7 | 5.58 | 5.12 |
| West Nusa Tenggara | 70.2 | 54.38 | 15.82 | 7.9 | 5.56 | 2.34 | 5.9 | 4.34 | 1.56 | 21.9 | 12.04 | 9.86 |
| West Papua | 54.9 | 24.81 | 30.09 | 75.4 | 25 | 50.4 | 34 | 11.6 | 22.4 | 73.7 | 23.66 | 50.04 |
| West Sulawesi | 52.8 | 31.24 | 21.56 | 15.5 | 5.53 | 9.97 | 11.6 | 2.78 | 8.82 | 20.6 | 5.22 | 15.38 |
| West Sumatra | 49.8 | 29.45 | 20.35 | 33.4 | 10.79 | 22.61 | 14 | 4.54 | 9.46 | 22.6 | 8.36 | 14.24 |
