# Supplementary material for: Using an Electronic Immunization Registry (Aplikasi Sehat IndonesiaKu) in Indonesia: Cross-Sectional Study
Source: Interact J Med Res. 2025 Mar 27;14:e53849. doi: 10.2196/53849 (PMC11986391; doi:10.2196/53849)
Supplement: Multimedia Appendix 4 [file ijmr_v14i1e53849_app4.docx]

Appendix 4

4.1 Bulan Imunisasi Anak Nasional coverage for measles-rubella (MR) vaccine; oral polio vaccine (OPV); inactivated polio vaccine (IPV); and diphtheria, pertussis, tetanus, hepatitis B, and Haemophilus influenzae type B (DPT-HB-Hib) vaccine using Aplikasi Sehat IndonesiaKu data

| **Province** | **Coverage in ASIK** | | | | | | | | | | | |
| --- | --- | --- | --- | --- | --- | --- | --- | --- | --- | --- | --- | --- |
|  | **Measles-rubella**  **Target: 95%** | | | **OPV**  **Target: 80%** | | | **IPV**  **Target: 80%** | | | **DPT-HB-Hib**  **Target: 80%** | | |
|  | **n** | **N** | **%** | **n** | **N** | **%** | **n** | **N** | **%** | **n** | **N** | **%** |
| Aceh | 93,127 | 1,444,337 | 6.5 | 26,452 | 177,947 | 14.9 | 5,930 | 219,788 | 3 | 7,282 | 184,475 | 4.0 |
| **Bali*** | N/A | 0 | N/A | **775** | **915** | **84.7** | **1,254** | **1,426** | **88** | **3,733** | **3,620** | **103.1** |
| Bangka Belitung Islands | 113,418 | 315,581 | 35.9 | 789 | 5,021 | 15.7 | 619 | 12,006 | 5 | 1,021 | 5,353 | 19.1 |
| **Banten** | **901,465** | **931,740** | **96.8** | **111,255** | **105,771** | **105.2** | **163,826** | **173,701** | **94** | **188,804** | **172,619** | **109.4** |
| Bengkulu | 168,839 | 425,461 | 39.7 | 1,994 | 9,157 | 21.8 | 2,149 | 13,637 | 16 | 2,228 | 10,737 | 20.7 |
| **Central Java** | **1,996,112** | **2,069,562** | **96.5** | **115,771** | **131,724** | **87.9** | **266,730** | **308,332** | **87** | **277,543** | **325,940** | **85.2** |
| Central Kalimantan | 233,127 | 573,815 | 40.6 | 2,857 | 58,389 | 4.9 | 1,892 | 95,243 | 2 | 2,821 | 58,076 | 4.9 |
| Central Sulawesi | 104,316 | 708,642 | 14.7 | 1,810 | 24,972 | 7.3 | 1,322 | 64,686 | 2 | 2,041 | 43,691 | 4.7 |
| DKI Jakarta | 534,480 | 715,786 | 74.7 | 37,502 | 172,905 | 21.7 | 35,952 | 323,658 | 11 | 85,750 | 110,738 | 77.4 |
| East Java | 1,797,337 | 2,352,409 | 76.4 | 31,286 | 114,284 | 27.4 | 46,239 | 218,636 | 21 | 52,123 | 252,555 | 20.6 |
| East Kalimantan | 344,166 | 764,046 | 45.1 | 658 | 26,294 | 2.5 | 747 | 67,544 | 1 | 1,252 | 23,448 | 5.3 |
| East Nusa Tenggara | 714,600 | 1,421,995 | 50.3 | 17,794 | 93,436 | 19.1 | 32,513 | 152,512 | 21 | 24,569 | 79,562 | 30.9 |
| Gorontalo | 95,323 | 247,819 | 38.5 | 3,203 | 28,952 | 11.1 | 1,866 | 54,428 | 3 | 2,628 | 17,525 | 15.0 |
| Jambi | 299,409 | 771,210 | 38.8 | 8,130 | 26,187 | 31.1 | 9,175 | 44,547 | 21 | 7,784 | 30,770 | 25.3 |
| **Lampung** | 1,220,678 | 1,812,491 | 67.4 | **49,833** | **37,827** | **131.7** | 98,810 | 132,971 | 74 | **64,043** | **71,504** | **89.6** |
| Maluku | 93,222 | 424,951 | 21.9 | 2,912 | 26,069 | 11.2 | 2,576 | 69,764 | 4 | 3,744 | 24,397 | 15.4 |
| North Kalimantan | 58,622 | 160,782 | 36.5 | 383 | 7,559 | 5.1 | 564 | 13,261 | 4 | 853 | 8,103 | 10.5 |
| North Maluku | 115,545 | 296,671 | 39.0 | 3,475 | 18,519 | 18.8 | 3,505 | 30,348 | 12 | 3,787 | 27,761 | 13.6 |
| North Sulawesi | 124,468 | 495,870 | 25.1 | 1,232 | 14,228 | 8.7 | 777 | 25,195 | 3 | 1,772 | 14,808 | 12.0 |
| North Sumatra | 1,914,822 | 3,900,143 | 49.1 | 82,225 | 134,007 | 61.4 | 93,760 | 245,537 | 38 | 64,593 | 125,068 | 51.6 |
| Papua | 72,368 | 792,523 | 9.1 | 951 | 118,227 | 0.8 | 1,814 | 167,678 | 1 | 1,294 | 194,510 | 0.7 |
| Riau | 561,149 | 1,913,264 | 29.3 | 5,250 | 93,454 | 5.6 | 5,317 | 136,905 | 4 | 5,738 | 115,042 | 5.0 |
| Riau Islands | 254,375 | 597,179 | 42.6 | 4,494 | 16,124 | 27.9 | 2,682 | 33,457 | 8 | 2,339 | 25,703 | 9.1 |
| South Kalimantan | 408,802 | 962,128 | 42.5 | 11,221 | 48,898 | 23.0 | 9,830 | 87,634 | 11 | 11,604 | 47,472 | 24.5 |
| South Sulawesi | 873,851 | 1,820,100 | 48.0 | 14,409 | 75,161 | 19.2 | 15,611 | 111,082 | 14 | 17,073 | 151,924 | 11.2 |
| South Sumatra | 1,006,696 | 2,150,006 | 46.8 | 28,684 | 46,745 | 61.4 | 29,721 | 87,972 | 34 | 24,829 | 36,397 | 68.2 |
| Southeast Sulawesi | 178,039 | 663,959 | 26.8 | 4,869 | 66,722 | 7.3 | 4,501 | 107,827 | 4 | 3,589 | 56,383 | 6.4 |
| Special Region of Yogyakarta* | N/A | 0 | N/A | N/A | 0 | N/A | 402 | 2,720 | 15 | 1,113 | 2,125 | 52.4 |
| West Java | 2,314,948 | 3,365,614 | 68.8 | 341,174 | 576,639 | 59.2 | 413,522 | 718,941 | 58 | 478,202 | 891,759 | 53.6 |
| West Kalimantan | 441,758 | 1,123,899 | 39.3 | 3,948 | 78,005 | 5.1 | 6,059 | 97,776 | 6 | 4,446 | 79,635 | 5.6 |
| West Nusa Tenggara | 668,588 | 1,229,440 | 54.4 | 7,190 | 129,413 | 5.6 | 5,840 | 134,692 | 4 | 9,673 | 80,321 | 12.0 |
| West Papua | 59,854 | 241,242 | 24.8 | 6,145 | 24,584 | 25.0 | 4,349 | 37,479 | 12 | 3,810 | 16,101 | 23.7 |
| West Sulawesi | 104,391 | 334,103 | 31.2 | 1,687 | 30,503 | 5.5 | 1,193 | 42,990 | 3 | 1,642 | 31,478 | 5.2 |
| West Sumatra | 433,162 | 1,470,926 | 29.5 | 8,265 | 76,602 | 10.8 | 5,621 | 123,916 | 5 | 7,381 | 88,300 | 8.4 |
| **National** | **18,301,057** | **36,497,694** | **50.1** | **938,623** | **2,595,240** | **36.2** | **1,276,668** | **4,158,289** | **30.7** | **1,371,104** | **3,407,900** | **40.2** |
| **province excluded from MR campaign* | | | | | | | | | | | | |

4.2 Bulan Imunisasi Anak Nasional coverage for measles-rubella (MR) vaccine; oral polio vaccine (OPV); inactivated polio vaccine (IPV); and diphtheria, pertussis, tetanus, hepatitis B, and Haemophilus influenzae type B (DPT-HB-Hib) vaccine using manual data

| **Province** | **Coverage in manual report** | | | | | | | | | | | |
| --- | --- | --- | --- | --- | --- | --- | --- | --- | --- | --- | --- | --- |
|  | **Measles-rubella**  **Target: 95%** | | | **OPV**  **Target: 80%** | | | **IPV**  **Target: 80%** | | | **DPT-HB-Hib**  **Target: 80%** | | |
|  | **n** | **N** | **%** | **n** | **N** | **%** | **n** | **N** | **%** | **n** | **N** | **%** |
| **Phase 1** | | | | | | | | | | | | |
| Aceh | 280,017 | 1,444,335 | 19.4 | 72,574 | 197,612 | 36.7 | 15,278 | 245,729 | 6.2 | 25,323 | 195,289 | 13 |
| North Sumatra | 2,861,376 | 3,900,139 | 73.4 | 100,769 | 134,007 | 75.2 | 134,979 | 245,537 | 55 | 76,852 | 125,068 | 61.4 |
| West Sumatra | 732,755 | 1,470,923 | 49.8 | 25,570 | 76,602 | 33.4 | 17,320 | 123,916 | 14 | 19,937 | 88,300 | 22.6 |
| Riau | 880,867 | 1,913,263 | 46 | 13,559 | 93,454 | 14.5 | 9,746 | 136,905 | 7.1 | 17,053 | 115,042 | 14.8 |
| Jambi | 625,363 | 771,209 | 81.1 | 18,175 | 26,187 | 69.4 | 29,625 | 44,547 | 66.5 | 23,888 | 30,770 | 77.6 |
| South Sumatra | 1,467,762 | 2,150,004 | 68.3 | 39,264 | 46,745 | 84 | 50,446 | 87,972 | 57.3 | 36,117 | 36,397 | 99.2 |
| Bengkulu | 294,555 | 425,461 | 69.2 | 1,828 | 9,157 | 20 | 2,506 | 13,637 | 18.4 | 3,207 | 10,737 | 29.9 |
| Lampung | 1,486,987 | 1,812,488 | 82 | 34,800 | 37,827 | 92 | 113,717 | 132,971 | 85.5 | 63,787 | 71,504 | 89.2 |
| Bangka Belitung | 190,785 | 315,580 | 60.5 | 1,416 | 5,021 | 28.2 | 1,700 | 12,006 | 14.2 | 2,479 | 5,353 | 46.3 |
| Riau islands | 427,688 | 597,177 | 71.6 | 6,572 | 8,061 | 81.5 | 7,087 | 10,989 | 64.5 | 8,236 | 9,005 | 91.5 |
| West Nusa Tenggara | 862,896 | 1,229,439 | 70.2 | 10,224 | 129,413 | 7.9 | 7,935 | 134,692 | 5.9 | 17,590 | 80,321 | 21.9 |
| East Nusa Tenggara | 947,474 | 1,421,992 | 66.6 | 21,949 | 89,116 | 24.6 | 41,145 | 171,991 | 23.9 | 29,129 | 82,035 | 35.5 |
| West Kalimantan | 657,317 | 1,123,898 | 58.5 | 6,556 | 78,005 | 8.4 | 10,420 | 97,776 | 10.7 | 8,510 | 79,635 | 10.7 |
| Central Kalimantan | 372,295 | 573,815 | 64.9 | 5,472 | 58,389 | 9.4 | 4,378 | 95,243 | 4.6 | 7,843 | 58,076 | 13.5 |
| South Kalimantan | 520,801 | 962,126 | 54.1 | 14,208 | 48,898 | 29.1 | 12,427 | 87,634 | 14.2 | 14,959 | 47,472 | 31.5 |
| East Kalimantan | 537,385 | 764,043 | 70.3 | 2,649 | 26,294 | 10.1 | 2,744 | 67,544 | 4.1 | 5,508 | 23,448 | 23.5 |
| North Kalimantan | 98,305 | 160,782 | 61.1 | 1,355 | 7,559 | 17.9 | 1,676 | 13,261 | 12.6 | 2,437 | 8,103 | 30.1 |
| North Sulawesi | 303,253 | 495,867 | 61.2 | 4,059 | 14,228 | 28.5 | 3,680 | 25,195 | 14.6 | 7,484 | 14,808 | 50.5 |
| Central Sulawesi | 404,300 | 708,641 | 57.1 | 7,716 | 24,972 | 30.9 | 9,846 | 64,686 | 15.2 | 11,289 | 43,691 | 25.8 |
| South Sulawesi | 1,733,978 | 1,820,098 | 95.3 | 32,734 | 75,161 | 43.6 | 34,398 | 111,082 | 31 | 44,124 | 151,924 | 29 |
| Southeast Sulawesi | 345,856 | 663,958 | 52.1 | 9,913 | 66,722 | 14.9 | 8,963 | 107,827 | 8.3 | 11,696 | 56,383 | 21.2 |
| Gorontalo | 141,000 | 247,818 | 56.9 | 4,909 | 6,843 | 71.7 | 2,811 | 11,955 | 23.5 | 4,011 | 17,670 | 22.7 |
| West Sulawesi | 176,502 | 334,102 | 52.8 | 2,562 | 16,576 | 15.5 | 2,752 | 23,763 | 11.6 | 4,119 | 19,956 | 20.6 |
| Maluku | 294,379 | 424,950 | 69.3 | 16,042 | 26,069 | 61.5 | 12,312 | 69,764 | 17.6 | 17,931 | 24,397 | 73.5 |
| North Maluku | 203,975 | 296,670 | 68.8 | 4,787 | 18,519 | 26.3 | 7,948 | 30,348 | 26.2 | 7,237 | 27,761 | 26.1 |
| West Papua | 132,466 | 241,241 | 54.9 | 18,527 | 24,584 | 75.40. | 12,751 | 37,479 | 34 | 11,865 | 16,101 | 73.7 |
| Papua | 307,466 | 792,523 | 38.8 | 13,754 | 118,227 | 11.6 | 5,521 | 167,678 | 3.3 | 9,676 | 194,510 | 5 |
| **Total of phase 1** | **17,287,803** | **27,062,542** | **63.9** | **491,943** | **1,464,248** | **33.6** | **564,111** | **2,372,127** | **23.8** | **492,287** | **1,633,756** | **30.1** |
| **Phase 2** | | | | | | | | | | | | |
| Jakarta | 710,757 | 715,786 | 99.3 | 34,155 | 172,905 | 19.8 | 30,088 | 323,658 | 9.3 | 82,867 | 110,738 | 74.8 |
| West Java | 3,221,028 | 3,365,611 | 95.7 | 483,781 | 503,611 | 96.1 | 611,346 | 664,213 | 92 | 735,141 | 822,143 | 89.4 |
| Central Java | 2,025,865 | 2,069,560 | 97.9 | 113,325 | 106,194 | 106.7 | 277,016 | 282,970 | 97.9 | 293,073 | 297,231 | 98.6 |
| DI Yogyakarta | N/A | 0 | N/A | N/A | 0 | N/A | 400 | 434 | 92.2 | 1,108 | 1,146 | 96.7 |
| East Java | 2,365,820 | 2,352,401 | 100.6 | 99,368 | 101,565 | 97.8 | 195,047 | 212,128 | 91.9 | 209,179 | 226,952 | 92.2 |
| Banten | 918,323 | 931,739 | 98.6 | 107,570 | 105,085 | 102.4 | 163,614 | 167,861 | 97.5 | 193,796 | 199,337 | 97.2 |
| Bali | N/A | 0 | N/A | 786 | 732 | 107.4 | 1,247 | 1,173 | 106.3 | 3,606 | 3,639 | 99.1 |
| **Total of phase 2** | **9,241,793** | **9,435,097** | **98** | **838,985** | **990,092** | **84.7** | **1,278,758** | **1,652,437** | **77.4** | **1,518,770** | **1,661,186** | **91.4** |
| **Total of phase 1 and 2** | **26,529,596** | **36,497,639** | **72.7** | **1,331,019** | **2,454,340.00** | **54.2** | **1,842,869** | **4,024,564** | **45.8** | **2,011,325** | **3,294,942** | **61** |
